# Validating a scale to measure engineers’ perceived self-efficacy for engineering education outreach

**DOI:** 10.1371/journal.pone.0223728

**Published:** 2019-10-16

**Authors:** Laura Fogg-Rogers, Tim Moss

**Affiliations:** 1 Science Communication Unit, University of the West of England, Bristol, England, United Kingdom; 2 Department of Engineering Design and Mathematics, University of the West of England, Bristol, England, United Kingdom; 3 Department of Psychology, University of the West of England, Bristol, England, United Kingdom; Aalborg University, DENMARK

## Abstract

Education outreach in schools has been identified as a critical route to influence children’s perceptions and capabilities for Science, Technology, Engineering, and Mathematics careers. Evidence suggests that providing non-teaching professionals like engineers with training programmes and structured experience can boost perceived self-efficacy to perform education outreach, which in turn means better quality and more frequent public engagement. A validated measure of the construct of perceived self-efficacy for engineering education outreach will be useful for effective science communication participation, research, and practise. This article presents the methods used to develop the Engineering Outreach Self-efficacy Scale (EOSS), along with initial reliability and validation results to support the scale’s use. The 10-item scale was found to have good internal consistency and reliability (Cronbach’s alpha α = .92) with a sample of 160 engineers. The scale had convergent validity with general self-efficacy. Engineers with more experience of education outreach had higher self-efficacy for engineering education outreach. There were no significant differences between male and female engineers. Initial test-retest results showed engineers receiving training in education outreach significantly improved their EOSS scores, indicating capability to detect change over time. It is hoped this scale will prove useful for further evaluation of engineering education outreach and public engagement with science activities.

## 1. Introduction

Education outreach is one form of public engagement whereby non-teaching professionals engage with young people, either in informal or formal learning environments [1,2]. Although often taking place in a formal educational context, education outreach programmes can share many of the characteristics of informal science learning opportunities, in that they are not bound by the constraints of the curriculum and school timetabling and can provide access to resources (people and equipment) which are not otherwise available in schools [3].

This is particularly important in Science, Technology, Engineering, and Mathematics (STEM) fields, with the aim of forming positive perceptions of these career paths as well as scientists or engineers themselves, in order to broaden children’s science capital [4]. Children with higher science capital have been shown to pursue higher STEM qualifications, and to work towards further STEM careers [5]. It has been argued that the informal learning sector is well placed to embed scientific ideas within a wider context [6], which is important for consolidating and contextualising learning [7]. Studies suggest that science outreach activities can increase interest and engagement with science and change pupils’ views of scientists [8], while teachers also value expert contributions to scientific knowledge [9].

Scientists and engineers are actively encouraged to voluntarily take part in public engagement with schools in the UK [10]. There is a UK Government programme of activity called ‘STEM Ambassadors’ which involves over 30,000 people from over 2500 companies running education outreach activities, which has shown positive benefits for both children and the professionals [11]. Indeed, education outreach is the most common form of public engagement with science activity which is reported by scientists and engineers in the UK [12]. Not all scientists and engineers who perform education outreach are registered as STEM Ambassadors, however, those that do are provided with brief training about the school system and a criminal records check. All these volunteers in schools are not provided with extensive training in pedagogy or formal learning environments, and are considered to be subject experts rather than education experts.

As more scientists and engineers voluntarily undertake education outreach, it is important to assess the preparedness of untrained professionals (in an educational context) to undertake this work, as well as the impact of these activities on the professionals themselves. This article uses the psychological construct of perceived self-efficacy (PSE), as a way to capture the beliefs people hold about their ability to succeed in an area of activity [13]. A validated measure of this construct for education outreach would therefore be useful for effective science communication training in order to develop best practice in the field. This paper therefore describes the development and validation of a scale to measure the latent construct of PSE pertaining to engineering education outreach.

### 1.1. Engineering education outreach

Education outreach has been identified as a critical area within the field of engineering, aiming to influence public perceptions and capabilities for the future engineering workforce [14,15]. Engineering constitutes 26% of the UK’s Gross Domestic Product, and yet there is an estimated shortfall of 20,000 engineering graduates every year, meaning that demand cannot currently be met [15]. Many public engagement efforts also aim to improve the diversity of entrants and graduates in engineering; in the UK only 12% of engineers are female, while 7% are from black and minority ethnic (BME) backgrounds [15] (compared to a BME population total of 14% [16]).

Engaging with engineers in person has been shown to improve children’s learning and attitudes towards engineering [17,18] along with stable (not declining) recruitment to local higher education engineering programmes [19]. Undertaking education outreach also benefits engineers themselves, enabling the mastery of generic skills such as communication and teamwork [2,20,21].

While professional bodies encourage engineers to take part in public engagement activities [22], participation depends on many factors. Studies with scientists undertaking public engagement show that feelings of enjoyment and personal efficacy, professional obligation, and a personal commitment to the public good are all critical [23]. On an organisational level, visible supportive leadership [24] and a culture whereby scientists and engineers consider public engagement as a normal and beneficial workplace activity are important [25,26].

As such, science communication literature indicates that it is becoming ever more important for enhanced training and support for public engagement programmes [27], as well as methods to measure their impact [28]. While much work exists to explore the impact of outreach on young people, there has only been one validated measure for assessing the outcomes for scientists or engineers themselves. The Self-efficacy for Public Engagement with Science scale measures scientists’ self-efficacy for taking part in public engagement activities [29]. A similar scale to measure engineers’ PSE for education outreach activities has the potential to enhance research into science communication training, as well as improve engagement practice for enhanced experiences for young people.

### 1.2. Perceived self-efficacy for engineering outreach

Perceived self-efficacy is a measure of self-belief in one’s own personal capabilities to produce specific actions. Bandura’s self-efficacy theory [13] suggests that if people believe that an action will have a favourable result and that they can successfully perform the action, they will be motivated to perform that action. The beliefs that individuals hold about their abilities and eventual outcomes are a powerful influence on how they will behave [30]. Self-efficacy is therefore a measure of a perceived ability, rather than actual performance, however, people with high PSE are more likely to continue performing that action [31]. People with high PSE can also influence others; research has shown that increasing a teacher’s confidence in their subject knowledge and their teaching self-efficacy (a belief that a teacher has in their ability to teach a subject well) has been associated with increased pupil achievement and motivation [32].

We therefore propose that the construct of engineering outreach self-efficacy is a belief in one’s own ability to explain engineering concepts in a way that will have positive outcomes for learners. PSE is specific for each activity [33], so while engineers may have high PSE for communicating with fellow engineers, they may have low PSE for undertaking education outreach activities with wider publics. PSE is in some way innate, in that some people will generally have a higher self-belief in themselves [34], however, PSE can be improved through personal experience. Bandura [30] identified four aspects which potentially contribute to the development of self-efficacy: mastery or performance accomplishments (i.e. experiences of relevant success); vicarious experiences (i.e. comparisons of capability to others, modelling and observing); verbal persuasions (positive feedback from peers and supervisors, coaching), and emotional arousal. This is why Dudo [35] asserts that science communication training is important to improve self-efficacy and therefore capacity for public engagement activities, including education outreach in formal education environments.

We argue in this paper that improving engineers’ self-efficacy is an important goal for public engagement and education outreach programmes. Researchers can assess self-efficacy beliefs by asking individuals to report on the strength of their confidence to accomplish or succeed in a task but it is important that reliable and relevant tools are used for the specific skill domain in question [33]. As such a validated scale for measuring engineers’ PSE for engineering education outreach will be useful to evaluate the impacts of these activities. The development of the scale has also provided further data on the demographics and personality traits of people engaging in engineering outreach in the UK.

## 2. Methods

The development of this Engineering Outreach Self-efficacy Scale (EOSS) was guided by recent reviews urging for more construct validity testing in psychology [36]. The instrument was developed using the self-efficacy scale guidelines from Bandura [33] as well as substantive item development to ensure content relevance and representativeness. The draft EOSS scale was then structurally analysed for reliability, as well as externally tested to determine convergent and discriminant validity.

### 2.1. Substantive scale development

The construct of EOSS was influenced by concepts outlined in the literature, describing the skills and challenges that can be perceived in the context of engineering education outreach. The item questions were also influenced by the personal experience of the lead author, through organising several engineering education outreach training programmes [2,37].

The instrument uses a 10-point Likert scale ranging from Not at All Confident in the ability to perform the item, to Totally Confident in the item. However, numbers represent the scale rather than words, as Bandura advises this wide range of options offers more discriminatory scalar analysis [33]. The wording of the questions was influenced by similar scales accessing PSE, and in particular the Teaching Engineering Self-efficacy Scale (TESS) [38]. While this scale measures a similar domain of self-efficacy, it is aimed at teachers instructing children in engineering, rather than engineers performing education outreach. As such, the scale was adapted for the specific skill domain of education outreach, as recommended by Bandura [33].

Initially, 15 questions were developed which aimed to assess aspects of PSE for engineering education outreach, including communicating subject knowledge, interacting with young people and their teachers/families, preparing for activities, connecting education outreach into wider academic or real world concepts, and perceived personal time and benefits of education outreach,. The questions were reviewed by members of the engineering department at the authors’ university, with initial discussions taking place at a department away day (with around 30 staff present). Three staff members who were experienced at engineering education outreach gave more thorough feedback on the potential questions using a written feedback form. This led to five questions being excluded as the questions were deemed to be beyond the realities of what was possible to personally control in an outreach environment. The final 10-item scale was reviewed for readability by the authors and the three staff members. In common with all PSE scales, the score for each item needs to be averaged across the whole 10-item scale in order to give the final mean PSE value for the scale.

### 2.2. Sample and procedure

Ethics Approval for the study was granted by the university Faculty Research Ethics Committee FET.17.02.025, and all participants gave informed consent. A total sample of 160 engineers were recruited to test the scale for internal and external reliability and validity. As the EOSS scale is intended to be used by any gender, age, or experience of engineer, a wide variety of engineers with varying demographic characteristics were recruited.

The total sample consisted of four convenience sample sub-groups. Undergraduate students at the authors’ university completed a paper-based version of the questionnaire before (*N =* 69) and after (paired *N =* 31) taking a module about Engineering and Society, providing training and experience in education outreach. Data were then transcribed into Microsoft Excel 2016 and cleaned for analysis in SPSS v24.

Another sub-group included professional engineers who self-identified as having undertaken engineering education outreach (N = 39) were also invited to complete a web-based version of the questionnaire (using Bristol Online Surveys) between September and November 2017. The engineers were recruited using emails, web notices and social media call-outs to professional networks of science communicators and engineers. The questionnaire was billed as finding out more about ‘who does engineering education outreach in the UK’.

Two final sub-groups of engineers recruited to the project were junior (*N =* 26) and senior (*N =* 26) female engineers taking part in the Women Like Me mentoring scheme at the authors’ university [39]. All 52 female engineers completed online questionnaires before the project, and 30 completed the paired post-project questionnaire. Data were downloaded into Microsoft Excel 2016 and combined with the student dataset to conduct descriptive and analytical statistics in SPSS v24.

### 2.3. External validity

All 160 participants completed a questionnaire which featured the EOSS questions. The student engineers and external professional engineers (*N =* 108) also completed additional validated questionnaires. In order to conduct an analysis of convergent validity, a known scale was selected for general self-efficacy, which was hypothesised to positively correlate with the results of the EOSS scale. The National Institutes of Health Toolbox v2.0 scale for Self-Efficacy (in 18+ adults) was used, as it is standardised and tested for use in the general population [40].

In order to provide an analysis of discriminant validity of the EOSS scale, the Mini-IPIP was used within the questionnaire as well [41]. This scale enabled a deeper understanding of the personality traits of engineers undertaking engineering education outreach, as well as any potential relationships between personality and Engineering Outreach Self-efficacy. The Mini-IPIP is a validated 20-item short form version of the International Personality Item Pool which assesses the Big Five Factor Model of personality including Conscientiousness, Extraversion, Agreeableness, Neuroticism and Openness/Imagination (this trait is called either Openness or Imagination in different studies, and so we have used both throughout this paper). It was hypothesised that there would be no significant correlation between personality traits and the scores for the EOSS, as personality is not linked to PSE in the literature.

## 3. Results

Multiple analyses of the data were conducted to describe the reliability and validity of the EOSS and to explore the responses of the test sub-samples. As the EOSS and comparative questionnaires utilise scalar data (and had a normative distribution), parametric statistical tests were used to analyse sub-sample and questionnaire responses.

### 3.1. Participants

The sample as a whole was 51% female, and consisted of 57% professional engineers, and so was fairly balanced. However, the sub-groups differed in their make-up, as can be seen in Table 1. The student sample was 9% female; a figure which reflects the low proportion of women engineers in the UK (currently at 12% [15]). The external professional sample was 59% female, which indicates a disproportionate completion of the questionnaire by women. Similarly, the Women Like Me sample was entirely female (by design).

**Table 1:** Characteristics of the test samples.

Demographic Characteristic		Whole sample % (*N = 160*, *% of this figure*)	Professional external sample % *(N = 39*, *percentages are calculated from this figure)*	Women Like Me sample % *(N = 52*, *percentages are calculated from this figure)*	Student sample % *(N = 69*, *percentages are calculated from this figure)*
Discipline	Aerospace Engineering	44	15	33	70
	Mechanical Engineering	5	10	10	0
	Electrical Engineering	8	10	6	10
	Biomedical Engineering	3	13	0	0
	Civil Engineering	14	15	25	0
	Other	14	15	19	20
Gender	Female	51	59	100	9
	Male	49	41	0	91
Age	16–24 years	37	5	10	83
	25–34 years	37	54	50	17
	35–44 years	21	18	29	0
	45–54 years	3	10	12	0
	55–64 years	2	8	0	0
	65+ years	1	3	0	0
Public engagement experience	Education outreach	64	90	70	37
	Science festival / open day expo	49	59	58	39
	Public talk/discussion	44	56	23	32

The professional engineers were recruited from a wide range of engineering disciplines as seen in Table 1, while 70% of the student engineers were taking Aerospace Engineering (a function of the module requirements). The student engineers were a younger sample as would be expected, but the professional engineers were more spread in their ages, with the majority (52%) being aged 25–34 years. The professional sample was also more experienced at public engagement activities, with 90% having taken part in education outreach, compared to only 37% of the students.

### 3.2. Exploring EOSS internal structure

The results for each item on the scale can be seen in Table 2. Each item had a wide range of responses which indicates good discriminant potential. The mean scale scores for the whole sample ranged from 2.8 to 10, with a Scale Mean of 6.63 (*SD =* 1.57), which indicates that the participating engineers had broadly high PSE for engineering outreach overall. An unpaired t-test indicated that there were no significant differences at the p < .05 level between how males and females scored on the EOSS, with the female mean score of 6.80 (*SD =* 1.64) being only slightly higher than the male mean score of 6.44 (*SD =* 1.48) [F (1,159) = 2.29, p = .132]. However, there was a significant difference between the student mean score of 6.24 (*SD* = 1.47) and the professional mean score of 6.91 (*SD =* 1.59) [F (1,159) = 7.46, p = .007], indicating that the professionals had higher overall PSE for engineering education outreach.

**Table 2:** Scores for each item on the Engineering Outreach Self-efficacy Scale.

Items on Engineering Outreach Self-efficacy Scale	Range for whole sample (Items are scored out of 10 and a high score indicates higher PSE)	Mean (*N = 160*)	Standard deviation	Factor Loading	Component Matrix Factor 1
1. I can discuss how engineering is connected to daily life and other subject areas.	1–10	7.42	1.81	.420	.858
2. I can plan out an engineering outreach activity which is engaging for young people.	1–10	6.00	2.19	.661	.839
3. I can spend the time necessary to plan engineering outreach activities.	1–10	6.21	2.13	.251	.823
4. I can communicate engineering concepts effectively to young people during outreach activities.	1–10	6.75	1.96	.677	.816
5. I can demonstrate engineering activities effectively in outreach sessions.	2–10	6.86	2.00	.736	.813
6. I can encourage young people to creatively explore ideas through the engineering design process.	1–10	6.61	2.03	.703	.801
7. I can judge young people’s comprehension of the engineering materials that I have presented.	1–10	6.42	1.90	.612	.782
8. I can inspire young people to enjoy engineering or wider scientific and mathematical concepts.	1–10	6.77	1.90	.641	.741
9. I can inspire teachers or parents to encourage young people’s interest in engineering or wider scientific and mathematical concepts.	1–10	6.69	2.12	.665	.648
10. I am confident that my efforts in engineering outreach are recognised and appreciated by peers in my professional environment.	1–10	6.28	2.52	.550	.501

Cronbach’s coefficient alpha was conducted as a basic measure of reliability [36], and indicated high internal consistency of the items (α = .92). There were no significant correlations between the items, and removing any of the items did not lower the Cronbach’s alpha below α = .91. This was repeated for both the student and professional sub-groups, and there were no significant correlations between items for the sub-groups either. A Kaiser-Meyer-Olkin Measure of Sampling Adequacy indicated that a Factor Analysis could be carried out (KMO = .92); this indicated that all the items loaded onto only one factor (see Table 2). Overall the scale was found to have a high internal reliability with a good discriminatory capacity for measuring differences between subjects in engineering education outreach PSE.

### 3.3. Exploring EOSS external validity

The EOSS scores were compared with established validated scales to see if the EOSS scale was measuring a valid and reliable construct of PSE for engineering education outreach; the mean scores can be seen in Table 3. The uncorrected mean score for the NIH Toolbox Self-Efficacy scale was 39.61 (*SD =* 4.19) which indicates that the engineers have higher than average general self-efficacy. A Pearson product-moment correlation coefficient test indicated that there was a significant positive correlation between general PSE and engineering outreach PSE r = .454, n = 108, p < .000. This shows that the more self-efficacious people feel in their general life, the more likely they are to score highly for engineering outreach PSE, and indicates high convergent validity for PSE.

**Table 3:** Associations between existing scales and the EOSS scores in this study.

Scale		Range	Mean score	Standard Deviation	Pearson Correlation between test score and EOSS
EOSS (*N = 160*) (Scale is scored out of 10 and a high score indicates higher PSE)		2.8–10	6.63	1.57	N/A
NIH Toolbox Self-efficacy *(N = 109)* (Uncorrected scale is scored out of 50 and a high score indicates higher PSE)		27–50	39.61	4.19	r = .454; p < .000*
Mini IPIP *(N = 109)* Scales are scored out of 5 with a lower score indicating a tendency towards this personality trait.	Openness/Imagination	1.0–5.0	2.20	0.91	r = -.126; p = .195
	Conscientiousness	1.25–4.75	2.50	0.78	r = -.115; p = .236
	Extraversion	1.0–4.75	2.84	0.87	r = -.086; p = .374
	Agreeableness	1.0–5.0	2.37	0.84	r = -.133; p = .171
	Neuroticism	1.0–5.0	3.51	0.80	r = -.009; p = .923

* Indicates significance at the p<0.05 level.

The Mini-IPIP scores indicated that the engineers differed widely in their personality traits. In general, the engineers tended towards high Openness/Imagination (*M =* 2.84 out of 5, where 3 is the middle, and a score towards 1 indicates a tendency towards this trait), Conscientiousness (*M =* 2.50, Extraversion (*M =* 2.84) and Agreeableness (*M =* 2.37) and tended towards low Neuroticism (*M =* 3.51). None of the personality traits had a significant correlation with EOSS as expected, which indicates good discriminant validity.

### 3.4. Personality traits of engineers participating in education outreach

The Mini IPIP scores were also analysed for significant differences and correlations between the whole sample personality traits, and between male/female and student/professional participants to further explore personality traits of engineers self-identifying as participating in education outreach. Pearson product-moment correlation coefficient tests indicated that there were significant differences at the p < .05 level. The NIH Toolbox Self-Efficacy scale was negatively correlated with Openness/Imagination (r = -.263, n = 108, p = .006), as well as with Conscientiousness (r = -.206, n = 108, p = .032). The personality trait of Openness/Imagination was positively correlated with the personality traits of Agreeableness and Conscientiousness (respectively: r = -.591, n = 108, p < .000; r = .276, n = 108, p = .004) and a negative correlation to Neuroticism (r = -.286, n = 108, p = .003). Agreeableness was significantly positively correlated with Extraversion and Conscientiousness (respectively r = .286, n = 108, p < .000; (r = .309, n = 108, p = .001), and there was a significant negative correlation with Neuroticism (r = -.330, n = 108, p < .000).

The two sub-samples differed slightly in their personality traits as well. A one-way between subjects ANOVA test indicated that there was a significant differences at the p < .05 level between students and professionals for Agreeableness, Neuroticism, and Openness. Students were significantly more likely to score highly on Agreeableness (*M =* 2.19) [F (1,108) = 9.24, p = .003] and Openness (*M =* 2.04) [F (1,108) = 5.36, p = .023] than professionals (Agreeableness *M =* 2.68 , Openness *M =* 2.46) and were significantly more likely to score low on Neuroticism (*M =* 3.74) (they were more emotionally stable) than professionals (*M = 3*.*13*); [F (1,108) = 16.56, p < .000].

A one-way between subjects ANOVA test was conducted for gender across the whole sample, and found that there were no significant differences at the p < .05 level between males and females for most of the tests including general self-efficacy. The only significant difference was in Neuroticism, whereby females tended more towards this trait (*M =* 3.20) than males (*M =* 3.63) [F (1,108) = 6.70, p = .011]; however, their scores were still low for Neuroticism.

### 3.5. Discriminant validity

Where possible the student engineers (*N =* 31) and Women Like Me engineers (*N* = 30) were re-recruited to retake the EOSS test after they had completed their programmes A paired-samples T-Test indicated that both sub-samples had their mean scores significantly increase. The collective sample (*N =* 61) significantly increased from *M =* 6.50 (*SD =* 1.55) to *M* = 7.94 (*SD* = 1.21); t(60) = -7.10, p < .000. The student sample increased from a score of 6.48 to 8.17 (t(30) = -5.99, = p < .000) and the junior professionals in the Women Like Me sample increased from a score of 6.60 to 8.41 (t(15) = -3.56, p = .003). Interestingly, the senior engineers did not receive training or experience in education outreach, and their scores on the EOSS did not significantly increase over time (t(13) = -1.28, p = .222). This indicates that the EOSS has good discriminant potential for showing change over time in PSE for engineering outreach.

## 4. Discussion

This study aimed to develop a self-efficacy scale to measure engineers’ PSE for engineering education outreach, along with providing initial validation evidence to support its use in practice. During the substantive phase [36], expert reviewers indicated that the construct has content relevance and representativeness. The internal validity tests indicated that the Engineering Outreach Self-efficacy Scale has structural integrity, with a full range of responses selected, and a high reliability for use, with Cronbach’s coefficient alpha α = .92. The EOSS has good convergent validity, with a significant correlation to the NIH Toolbox Self-Efficacy scale as hypothesised. This indicates that the more self-efficacious people feel in their general life, the more likely they are to score highly for engineering outreach PSE. While the total sample size of 160 engineers was relatively small, the results still showed that the EOSS also has good discriminant validity, with no significant correlations to personality factors on the Mini IPIP. Initial test-retest use indicated that the scale can discriminate change over time, as it detected a significant improvement in PSE for engineering outreach for the student and junior engineers (*N = 47*) who had received training and experience in education outreach.

### 4.1. Women ambassadors as role models for engineering

Testing the EOSS also provided an opportunity to find out more about engineers taking part in engineering education outreach in the UK. The student engineers reflected the general student engineering population, however, the self-selecting professional engineers included a higher proportion of women engineers than national averages (59% in the sample versus 11% nationally). This may be due to recruitment bias in terms of who completed the survey, but it does reflect previous research which indicates that women are more likely to become involved in public engagement activities with children [42]. One of the sub-samples was recruited through the Women Like Me project, and so they were 100% female by design [39].

Indeed, 40% of STEM Ambassadors of female, although this figure includes scientists as well as engineers [43]. The higher proportion of women acting as Ambassadors than are present within the STEM workforce echoes previous research calling for more women to undertake public engagement in order to act as role models for girls interested in STEM careers [44,45]. The science communication literature discusses this dilemma; female role models in STEM are needed in order to broaden implicit societal messaging about which careers are appropriate for girls [46]. However, the over-representation of women undertaking engineering education outreach means that these activities may become burdensome for female engineers in their careers. Previous studies have indicated that undertaking public engagement can be perceived as a “soft option” that reduces STEM research career trajectories [23]. Further research with a larger sample size is needed to determine if a higher proportion of women undertake engineering education outreach, perhaps in conjunction with engineering professional bodies.

### 4.2. Engineers’ personality traits and self-efficacy

Overall, the engineers in this study had high general self-efficacy and moderately high PSE for engineering education outreach. They tended towards high Openness/Imagination, Conscientiousness Extraversion, and Agreeableness, and tended towards low Neuroticism. The range of personality traits demonstrated in the sample was very broad, which reinforces previous studies describing the wide variety of personality types working in engineering [47]. Many of the personality traits correlated together, which emphasises personality psychology research underlining general factors of personality that govern the five-factor model [48]. However, the personality profile represented in the sample does indicate a profile of engineers who are socially capable, as would be expected from those who voluntarily undertake education outreach.

The student sub-sample scored significantly higher than the professional sub-sample for Openness/Imagination and Agreeableness, and significantly lower for Neuroticism. One explanation for this may be that younger people, such as students, have less responsibilities to constrain them and make them neurotic, and professionals (who tended to be older) become more set in their ways as they age so less open to experience. However, this contradicts the established concept of personality tests measuring stable characteristics of a person, as most student engineers will eventually become professional engineers and so we should not see differences between student and professional populations. However, Feldt et al., [48] argue that there is nothing in the five-factor model which states that traits are fixed over time.

### 4.3. Public engagement experience

The professional sub-sample scored significantly higher on the EOSS than the student sub-sample, and also were more experienced at public engagement, with 90% having undertaken education outreach, as opposed to 37% of students. However, there were no significant differences in scores between men and women. This reinforces the idea that PSE can be improved through experience of success (mastery) and social modelling [31]. Furthermore, it provides additional evidence for the expansion of public engagement training programmes for scientists and engineers [35], with the emphasis on peer support and structured experiences to improve their self-efficacy for education outreach. Indeed, the EOSS was able to detect change over time, with the engineers who had received training in engineering outreach and undertaken structured programmes of outreach, subsequently scoring significantly higher on the scale. People with higher PSE are more likely to continue performing the specific activity, and so boosting self-efficacy for education outreach should see improvements in public engagement participation and practise.

### 4.4. Evaluating education outreach training programmes

This study has validated a new Engineering Outreach Self-efficacy Scale for use with engineers participating in education outreach. The sample size and statistical analyses are sufficient to conclude that the scale is reliable and valid, and can offer discriminant potential for change over time. Measuring engineering education outreach PSE provides a way to evaluate and compare the impact of outreach training programmes, and as such, we hope this scale will prove valuable to researchers and participants. Further use of this scale will enable wider quantitative comparisons of change in PSE for education outreach through providing science communication or public engagement training.

Evidence suggests that training programmes and structured experience can boost perceived self-efficacy for engineering education outreach, which in turn means that engineers may take part in better quality and more frequent public engagement. Enhancing the capacity and self-efficacy for engineers to undertake more education outreach and public engagement means they are more visible to act as positive role models for young people. Enriched education experiences will therefore benefit both children’s and teachers’ perceptions and capabilities for STEM qualifications and careers. This is a living work, and as such, the EOS construct needs to develop through expanded and continual use of this scale.
